# Chung Un Lee: the trailblazer of tropical medicine in China

**DOI:** 10.1093/procel/pwac046

**Published:** 2022-10-27

**Authors:** Huan Liu, Yifan Wei, Wanying Gao, Hao Cheng

**Affiliations:** University of Science and Technology of China, Hefei 230026, China; State Key Laboratory of Virology, Wuhan 430072, China; University of Science and Technology of China, Hefei 230026, China; University of Science and Technology of China, Hefei 230026, China; Institute of Microbiology, Chinese Academy of Sciences, Beijing 100101, China

Chung Un Lee, (李宗恩, 1894–1962) was a pioneer of tropical medicine research in China, serving as the president of Guiyang Medical College, the dean of Peking Union Medical College, the vice president of the Chinese Medical Association, a member of the British Medical Association, and the Far Eastern Tropical Medical Association (Fig. 1) (Lu, 1992). Chung Un Lee spent his life in medical education and scientific research, with outstanding achievements in research on filariasis, schistosomiasis, malaria, and kala-azar, confirming the reservoir of filariasis in China, innovating the application of plasmochin to combat malaria, proving that sandflies and dogs are sources of infection in human kala-azar, and making a clinical diagnosis of the early manifestations of kala-azar in China. Chung Un Lee is a distinguished medical scientist and medical educator, one of the founders of Chinese Tropical Medicine, who fought for 40 years for the prevention and treatment of tropical diseases in China and conducted pioneering research in Chinese Tropical Medicine.

**Figure 1.**
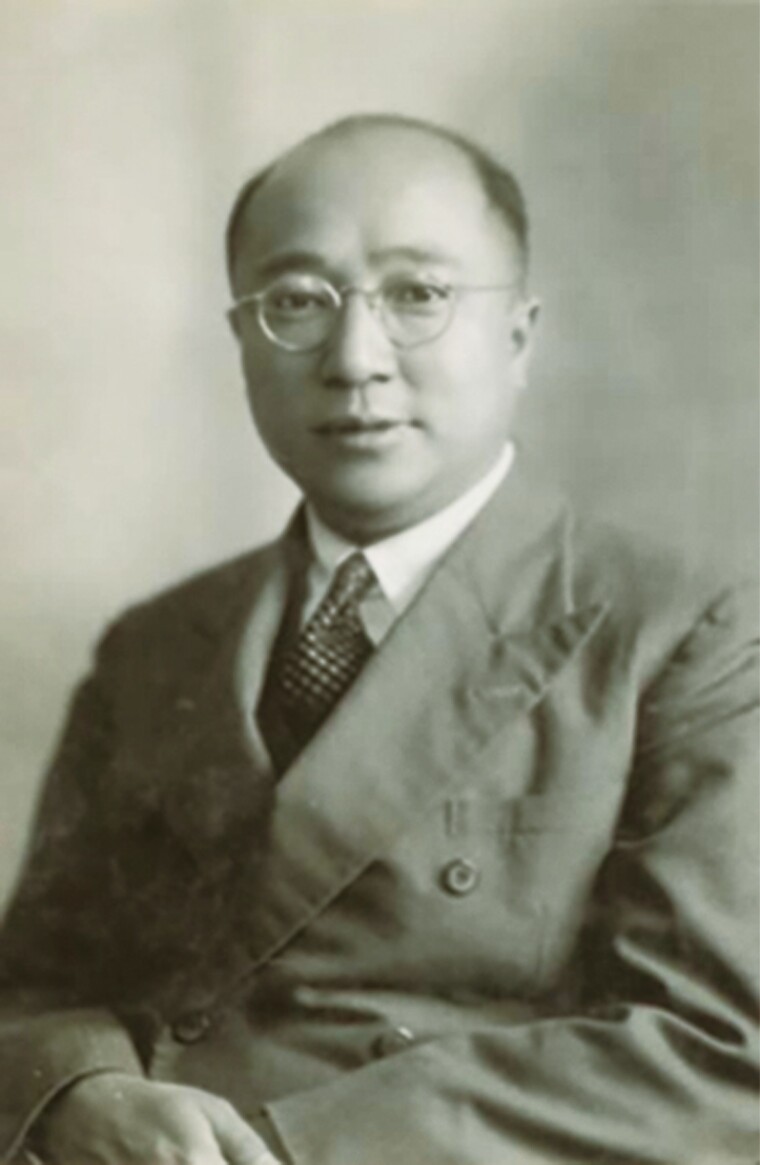
Chung Un Lee.

## Biography

**Figure 2.**
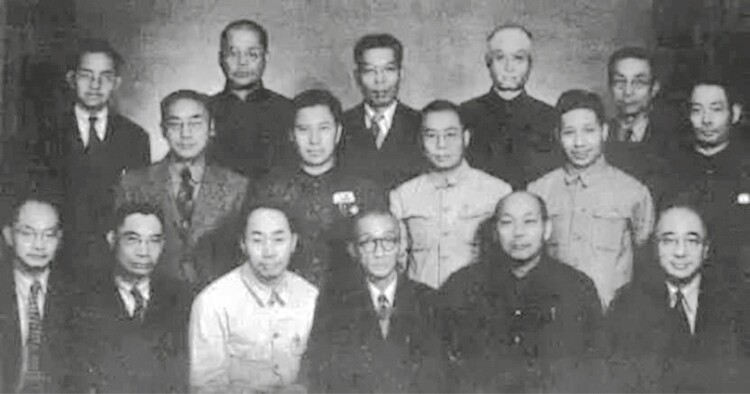
Attendees of the 1948 National Natural Scientists Congress of China (Chung Un Lee is the sixth from left in the first row).

Chung Un Lee, a native of Changzhou, Jiangsu Province, was born in 1894. His father was influenced by the Reform thoughts when he was the magistrate of Shandong and established the first local new-style primary school, where Chung Un Lee studied (Li, 2021). In 1908, Chung Un Lee went to Shanghai Aurora University to study. He went to the UK to study in 1911, was admitted to the medical school of the University of Glasgow in 1913, and received bachelor of medicine and bachelor of chemistry degrees in 1920. In 1922, he received a master’s degree in tropical diseases and hygiene from the London School of Hygiene and Tropical Diseases, University of London (Liao, 1992). In 1923, he returned to China to work at the Peking Union Medical College, and in 1938, he became the president of the National Medical College of Guiyang. In 1947, he became the first Chinese president of Peking Union Medical College. He was elected as an official delegate to the First Plenary Session of the Chinese People’s Political Consultative Conference in July 1948 (Fig. 2). He was the chairman of the European-American Association in 1949. From 1947 to 1957, he served as the president of Peking Union Medical College. In 1957, he went to Kunming Medical College for research work and died in Kunming in 1963 (Lu, 1992).

## Studying period

**Figure 3.**
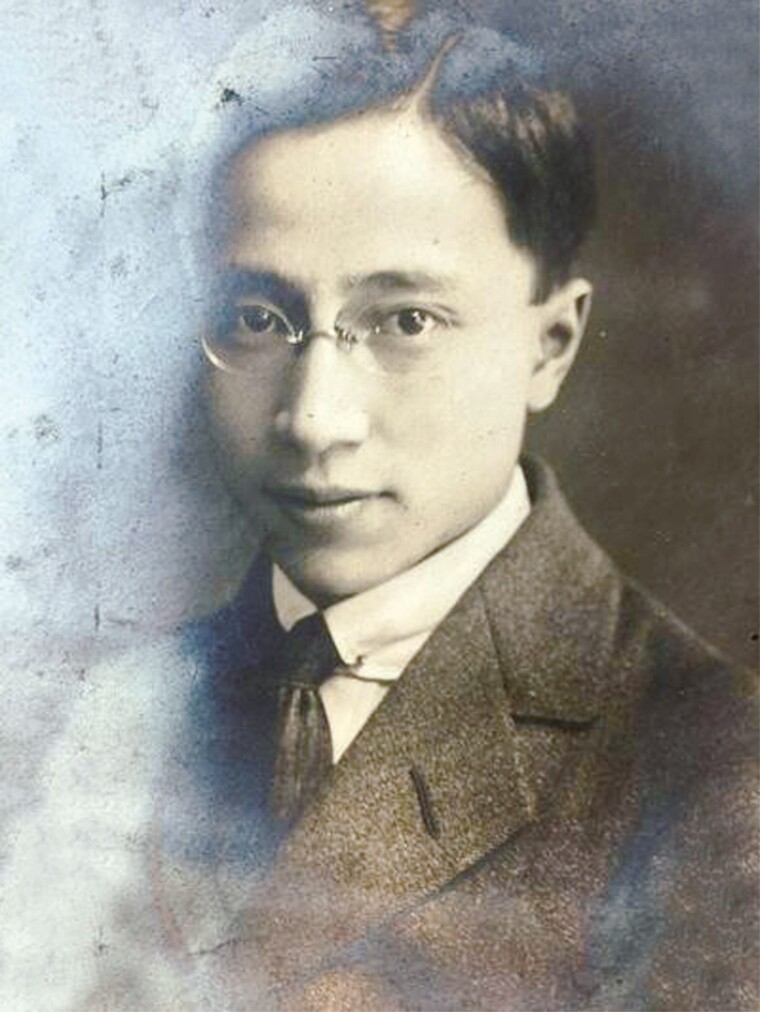
Chung Un Lee at the University of Glasgow.

In 1913, Chung Un Lee entered the medical school of the University of Glasgow in the UK (Fig. 3). The school’s archives retain the records of Chung Un Lee’s second prize in clinical internal medicine and the 13th place based on grades during his studies. In 1920, Chung Un Lee graduated from the University of Glasgow, England, with bachelor’s degrees in medicine and chemistry, and then went to the London School of Hygiene and Tropical Diseases, University of London, as an assistant researcher in helminthology. In April 1921, Chung Un Lee went to British Guiana with Robert Lepper’s five-member expedition from the London School of Tropical Medicine to investigate and study helminthic diseases. In January 1922, he graduated from the London School of Hygiene and Tropical Diseases, University of London, with a master’s degree in Tropical Medicine and Hygiene.

In 1923, Chung Un Lee published the article “A Filarial Survey in British Guiana, 1921” in the Journal of Helminthology, which included a survey visit to the local domicile, and covered ~50% of the Chinese in Georgetown. According to the census returns for 1921, there were 2,722 Chinese in British Guiana, mostly native-born, and the local Chinese were mostly spared from filariasis or elephantiasis. He found through blood tests and a survey of lifestyle habits that the lower incidence of filariasis among the Chinese was attributable to their attention to personal hygiene, with most falling asleep soon after dark and almost all sleeping under mosquito nets, a traditional habit that allowed them to avoid infection. This finding suggested that filariasis is dangerous regardless of race and that there is no racial immunity (Anderson et al., 1923).

In June 1923, Chung Un Lee returned to his homeland and took up an assistant professorship in the Department of Internal Medicine at Peking Union Medical College, from which he started a new medical career. Chung Un Lee pioneered the research of tropical diseases in China and established tropical disease observatories in the epidemic areas in the south. Many scientific studies from the 1920s and 1930s were focused on parasitic diseases, especially filariasis, malaria, schistosomiasis, and kala-azar.

## Prove *Culex pipiens* is the carrier of filariasis in China

**Figure 4.**
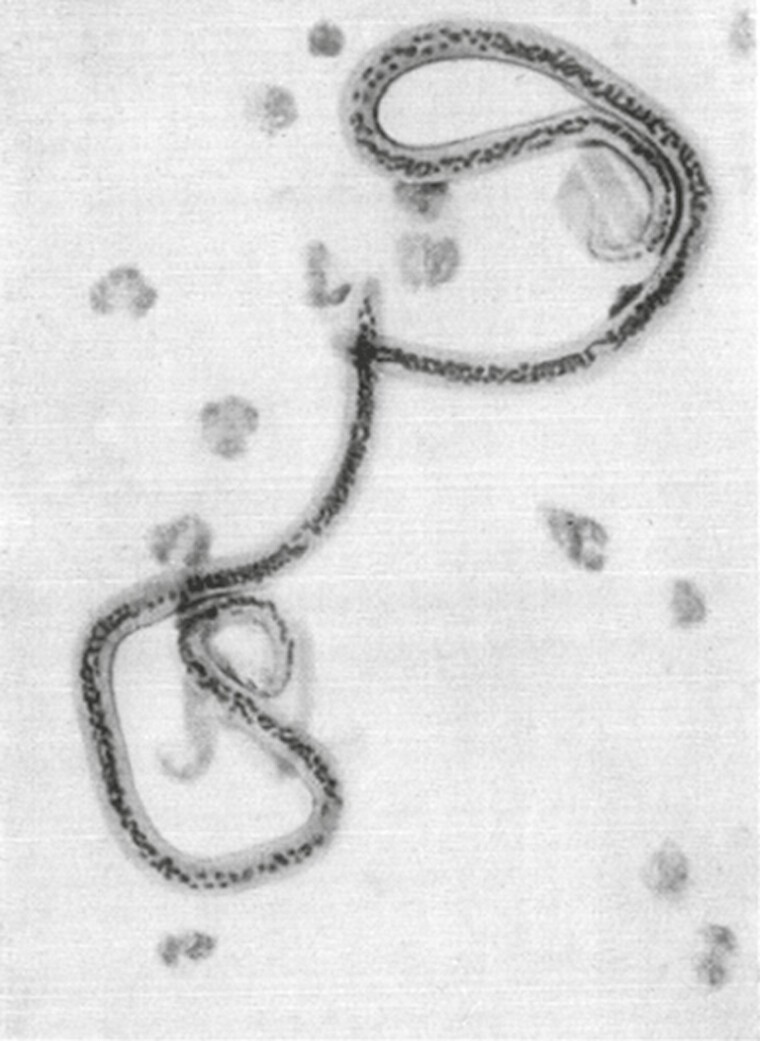
Microfilariae from the peripheral blood of an infected person.

At the beginning of the 20th century, there was high incidence of filariasis in China, which is typical in southern regions. In 1926, Chung Un Lee started his research on filariasis in China, and in response to the occurrence of filariasis and elephantiasis in the population north of the Yangtze River, Chung Un Lee examined 363 people for microfilament disease, finding that the rate of microfilament disease in people with signs of filarial infection was much higher than in those who were free from filarial disease (Fig. 4). He also found that the rate of *Microthrix parvicella* infection was high, whereby lymphatic disease is also evident. His study of paraffin sections of a series of mosquitoes collected from Tsing Kiang Pu revealed that a significant proportion of *C. pipiens* were negative for different stages of pathogen development, and although no larva was found in the rostrum, larvae at different stages of development were found in the pectoral muscles, which provides strong evidence that both *C. pipiens* and *Culex fatigans* can serve as effective intermediate hosts for *Filaria bancrofti* in China. This led to the conclusion that filariasis is carried by human-associated mosquitoes such as *Culex* spp., and is mainly caused by indoor infection (Lee et al., 1926). Through a series of experiments, C.U. Lee proposed methods to prevent filariasis by taking mosquito control measures and preventing mosquito bites. At that time, mosquito prevention measures such as land drainage, installation of piped water supply systems and sewage systems were almost impossible in China, but the use of mosquito nets could be encouraged for a good preventive effect. Mass education of the population about the dangers of mosquito bites due to the spread of diseases would have greatly reduced the incidence of malaria and filariasis in the country.

## Apply plasmochin to fight malaria

Chung Un Lee conducted extensive research on malaria, studying the identification, habits, and control of *Anopheles*, the vector of malaria, and proposing countermeasures for the epidemic and clinical treatment of malaria. In the Peking Union Medical College Hospital, Chung Un Lee’s team conducted >3,700 experiments in the decade from 1925 to 1935. In 1936, he proposed that two infections, malaria, and relapsing fever, occur after blood transfusion. Based on a study of the occurrence of 54 cases, he found that malaria in most people is the result of direct blood transmission from transfusion donors, thus proposing that blood transfusion is a route for transmitting the pathogen. Accidental transmission of benign tertian malaria or relapsing fever to a person whose health is already compromised by some existing disease is a risk factor for complications, and in malaria-endemic areas, the effects can even be catastrophic, so that the most stringent measures must be used to minimize the incidence of accidental transmission through blood transfusion (Wang et al., 1936).

In the 1920s, Chung Un Lee confirmed the value of plasmochin as an antimalarial drug through the treatment history of 8 cases of malaria, 6 cases of benign malaria, and 2 cases of aestivo-autumnal malaria in Beijing. Although the value of quinine and its associated alkaloids is undeniable, in many respects they are not ideal specific drugs. Quinine is more specific for aestivo-autumnal malaria and has little effect on clearing *Plasmodium falciparum* or crescents from the blood. Quinine’s strong bitter taste and induction of intoxication symptoms, especially in susceptible individuals, also greatly reduce its value as an antimalarial agent. In addition, the cultivation of quinine-derived cinchona trees is limited to certain regions of the world, and quinine production was monopolized by private companies, which further contributed to unreasonably high drug prices. It is out of these considerations that Chung Un Lee introduced plasmochin, a new synthetic product invented by the Elberfield Pharmaceutical Laboratory in 1925. It belongs to the same class of drugs as quinine, and treatment with a combination of quinine and plasmochin is more effective. In some cases of malaria, in which a decision needs to be made quickly, Chung Un Lee advocated the use of spleen puncture as a diagnostic tool. Even though some so-called irritant measures were applied in some cases, repeated blood examination failed to detect parasites, while after spleen puncture, there was a febrile reaction and parasites were found in peripheral blood (Lee, 1929).

## Action of organic antimony compounds on *Schistosoma*

Schistosomiasis is also a tropical disease with a high incidence in China, mostly in the Yangtze River basin, which is the hardest hit. Chung Un Lee devised a simple technical method that keeps adult schistosomes alive without difficulty in artificial media for several weeks, or even months if necessary. The viability of the worms varies in different media, which may depend more on the inherent vitality of the parasite than on the nature of the medium (Lee et al., 1935c). Chung Un Lee found that the life of adult helminths cultured *in vitro* could be extended up to two and a half months if the medium was changed frequently. This could be achieved using the small tissue culture method, which prevented bacterial contamination and desiccation of the media. Later, he studied the lethal action of four antimony compounds on schistosomes *in vitro*: chemically pure, sodium antimony tartrate, Fouadin, and antimony III-pyrocatechin-disulphonate of sodium (Lee et al., 1935a).

## Confirm sandflies and dogs as vectors of kala-azar

**Figure 5.**
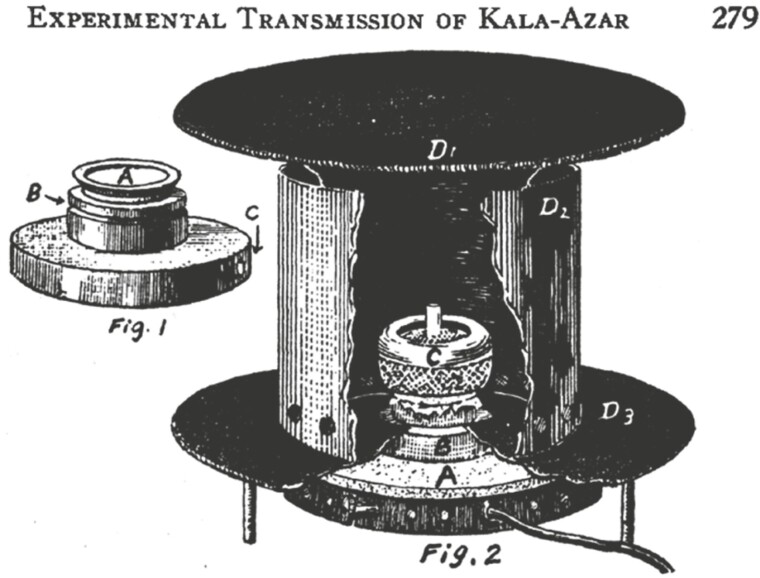
Aluminum cage for feeding sandflies.

In the 1930s, kala-azar was widely prevalent in China in the region north of the Yangtze River, with hundreds of thousands of patients. Chung Un Lee proposed that sandflies might be the vector of kala-azar. He used an artificial feeding device (Fig. 5) to squeeze the sandflies by tapping the itchy skin where the sandflies were feeding, mimicking the natural conditions of sandflies feeding and crushing as much as possible, although the experimental process was quite time-consuming because the sandflies had to be fed and tapped one after another. The results of this study confirmed that the sandflies can be infected by bites or human swats, that the breeding season of the sandflies coincides with the prevalence of kala-azar, and that cysts of kala-azar can mature and reproduce in sandflies (Hu and Lee, 1929; Hu et al., 1929).

Chung Un Lee’s research confirmed the infection of dogs with kala-azar and that patients could be infected by dogs. He conducted studies on the association of kala-azar in humans with sandflies and dogs, and identified the key factor of dogs in the epidemic of kala-azar. He and Dr Huei-Lan Chung confirmed that humans can be infected by dogs with kala-azar, and pointed out that the infection rate of dogs is much higher than that of humans after the bites of sandflies, thus concluding that dogs are the most important hosts of kala-azar (Chung et al., 1939). Based on these, many measures were developed after 1950 to eradicate the Chinese sandflies and cull dogs to eradicate kala-azar in humans. After years of efforts, kala-azar became almost extinct in most endemic areas of China, which became an outstanding achievement of medicine and disease prevention in China.

## Early manifestations of kala-azar

In a study of clinical manifestations and therapeutic drugs for kala-azar, Chung Un Lee conducted a clinical study of 394 cases of pathogenically confirmed kala-azar admitted to Peking Union Medical College Hospital, 51 of which were in the early stages. The results of the study showed that children are relatively susceptible to kala-azar, and the superficial lymph glands of children are significantly enlarged in the early stages of the disease. Severe complications may occur in the early stages of kala-azar, with two cases of acute agranulocytosis. Early diagnosis can be difficult since splenomegaly, leucopenia, and globulin precipitation are absent or negative. Early kala-azar must be differentiated from tuberculosis, malaria, typhoid fever, or undulant fever, and the possibility of pathogenesis can be considered when accompanied by a definite, progressive leucopenia-induced fever (Lee et al., 1935b). In his initial diagnosis of early kala-azar, he suggested: “The initial onset of kala-azar is latent, gradual, or sudden. The most common manifestations of kala-azar are slight feverishness, weakness, chills, night sweats, anorexia, headache, cough, constipation, slight loss of weight, diarrhea, epistaxis, abdominal distention, and runny nose. In the initial stage, sometimes there is no abnormality except weakness or anorexia and diarrhea, so the diagnosis is not possible. Even when fever and other symptoms are obvious, blood examinations and physical examinations are normal, and it is necessary to repeatedly depend upon spleen puncture or liver puncture, and take tests of the bone marrow and lymph gland before the diagnosis can be made.” (Chung et al., 1934).

In 1934, Chung Un Lee compared the efficacy of urea-stibamine and neostibosan in the treatment of kala-azar and suggested that urea-stibamine is more effective, but its toxicity is also more severe (Qu et al., 1934). In the study of tropical diseases, Chung Un Lee’s research on kala-azar with his colleagues at the Peking Union Medical College was considered “a successful example of Chinese and foreign medical scientists working together and applying modern scientific methods to study epidemic diseases in China.” He published a total of 18 papers in domestic and international medical journals. Chung Un Lee’s papers on malaria, filariasis and other epidemics published in tropical disease research journals have had a significant impact at home and abroad.

## Founder of Guiyang Medical College

**Figure 6.**
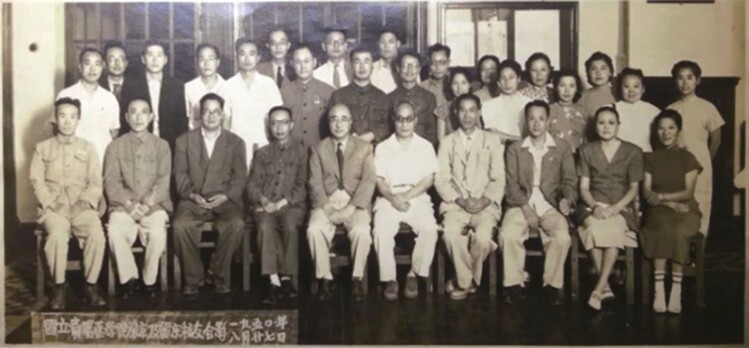
National Guiyang Medical College in 1950 (Chung Un Lee is the fifth from left in the first row).

In November 1937, Chung Un Lee left Beijing to attend the National Medical College of Guiyang, which was planned by the Ministry of Education to receive medical students affected by the war. On March 1 of the following year, the National Guiyang Medical College was established, and Chung Un Lee was officially appointed its president. Chung Un Lee’s nine years at Guiyang Medical College were also nine significant years for medical education in China (Fig. 6).

## First Chinese President of Peking Union Medical College

**Figure 7.**
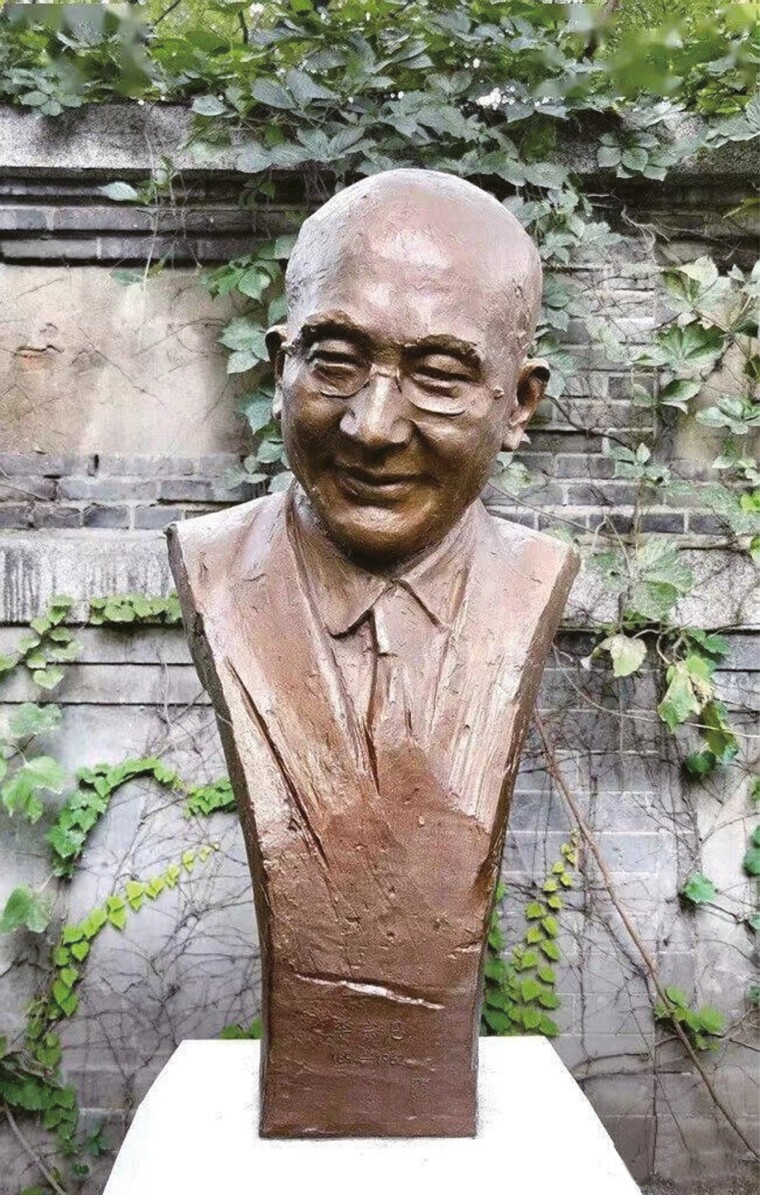
Sculpture of Chung Un Lee in Peking Union Medical College.

In 1947, Chung Un Lee was appointed president of the Peking Union Medical College, the first Chinese president in its history (Fig. 7). He firmly advocated the “eight-year medical education system” and trained excellent medical workers. Professor Jiadong Deng of the Peking Union Medical College once commented that he had “a wide knowledge of internal medicine, not limited to tropical medicine, and was a highly regarded Chinese teacher at the school.”

## Pioneer of tropical medicine in China

Chung Un Lee spent his life fighting for the eradication of tropical diseases in China, blazing the trail for research on kala-azar, filariasis, malaria, and schistosomiasis. He made various breakthrough achievements in tropical medicine and laid the foundation for related research in China. Chung Un Lee devoted himself to medical education, serving as the first president of the National Guiyang Medical College, where he proposed the motto of “Sincerity in oneself, loyalty to the group, and respect for the past and the future,” sending a large number of medical experts to China during the war. He served as the president of Peking Union Medical College, was elected as the first academician of Academia Sinica, was a member of the first national committee of the Chinese People’s Political Consultative Conference, and the chairman of the European and American Association. Chung Un Lee was a man who cared about the fate of the motherland and the suffering of the people, always adhering to the spirit of science and the principle of academic primacy, which made him a trailblazer in the research of tropical diseases in China.
